# Prevalence *of Plasmodium falciparum* delayed clearance associated polymorphisms in adaptor protein complex 2 mu subunit (*pfap2mu*) and ubiquitin specific protease 1 (*pfubp1*) genes in Ghanaian isolates

**DOI:** 10.1186/s13071-018-2762-3

**Published:** 2018-03-12

**Authors:** Tryphena Adams, Nana Aba A. Ennuson, Neils B. Quashie, Godfred Futagbi, Sena Matrevi, Oheneba C. K. Hagan, Benjamin Abuaku, Kwadwo A. Koram, Nancy O. Duah

**Affiliations:** 1 Department of Animal Biology and Conservation Science, School of Biological Sciences, College of Basic and Allied Sciences, University of Ghana, Accra, Ghana; 2 Department of Epidemiology, Noguchi Memorial Institute for Medical Research, College of Health Sciences, University of Ghana, Accra, Ghana; 3 Centre for Tropical Clinical Pharmacology and Therapeutics, School of Medicine and Dentistry, College of Health Sciences, University of Ghana, Accra, Ghana; 4 Department of Biochemistry, Cell and Molecular Biology, School of Biological Sciences, College of Basic and Allied Sciences, University of Ghana, Accra, Ghana

**Keywords:** *Plasmodium falciparum*, Antimalarial drug resistance, Artemisinin, ACT, *pfubp1*, *pfap2mu*, Mutations, Ghana

## Abstract

**Background:**

*Plasmodium falciparum* delayed clearance with the use of artemisinin-based combination therapy (ACTs) has been reported in some African countries. Single nucleotide polymorphisms (SNPs) in two genes, *P. falciparum* adaptor protein complex 2 mu subunit (*pfap2mu*) and ubiquitin specific protease 1 (*pfubp1*), have been linked to delayed clearance with ACT use in Kenya and recurrent imported malaria in Britain. With over 12 years of ACT use in Ghana, this study investigated the prevalence of SNPs in the *pfap2mu* and *pfubp1* in Ghanaian clinical *P. falciparum* isolates to provide baseline data for antimalarial drug resistance surveillance in the country.

**Methods:**

Filter paper blood blots collected in 2015–2016 from children aged below 9 years presenting with uncomplicated malaria at hospitals in three sentinel sites Begoro, Cape Coast and Navrongo were used. Parasite DNA was extracted from 120 samples followed by nested polymerase chain reaction (nPCR). Sanger sequencing was performed to detect and identify SNPs in *pfap2mu* and *pfubp1* genes.

**Results:**

In all, 11.1% (9/81) of the isolates carried the wildtype genotypes for both genes. A total of 164 *pfap2mu* mutations were detected in 67 isolates whilst 271 *pfubp1* mutations were observed in 72 isolates. The majority of the mutations were non-synonymous (NS): 78% (128/164) for *pfap2mu* and 92.3% (250/271) for *pfubp1*. Five unique samples had a total of 215 *pfap2mu* SNPs, ranging between 15 and 63 SNPs per sample. Genotypes reportedly associated with ART resistance detected in this study included *pfap2mu* S160N (7.4%, 6/81) and *pfubp1* E1528D (7.4%, 6/81) as well as D1525E (4.9%, 4/81). There was no significant difference in the prevalence of the SNPs between the three ecologically distinct study sites (*pfap2mu: χ*^2^ = 6.905, *df* = 2, *P* = 0.546; *pfubp1*: *χ*^2^ = 4.883, *df* = 2, *P* = 0.769).

**Conclusions:**

The detection of *pfap2mu* and *pfubp1* genotypes associated with ACT delayed parasite clearance is evidence of gradual nascent emergence of resistance in Ghana. The results will serve as baseline data for surveillance and the selection of the genotypes with drug pressure over time. The *pfap2mu* S160N, *pfubp1* E1528D and D1525E must be monitored in Ghanaian isolates in ACT susceptibility studies, especially when cure rates of ACTs, particularly AL, is less than 100%.

**Electronic supplementary material:**

The online version of this article (10.1186/s13071-018-2762-3) contains supplementary material, which is available to authorized users.

## Background

Malaria is still a debilitating disease, especially in sub-Saharan Africa (sSA) where there were 212 million cases and 429,000 malaria-related deaths in 2015 [1]. There has been a 21 and 29% reduction in morbidity and mortality, respectively, since 2010, probably as a result of the implementation of integrated control strategies [1, 2]. The control efforts employed included the use of insecticide-treated mosquito nets (ITNs), indoor residual spraying (IRS), chemoprevention in pregnant women and children as well as chemotherapy with artemisinin-based combination therapy (ACT). As such, the development of *Plasmodium falciparum* resistance to artemisinin (ART) derivatives as reported from Southeast Asia (SEA) is quite worrying [1]. Chemotherapy, which is one of the core control strategies for the disease, has been hindered over the years by the emergence and spread of parasites resistant to the commonly used antimalarial drugs [1]. Currently, the World Health Organisation (WHO) has initiated the containment of drug resistance in the SEA region with the deployment of a multi-sector strategy [1]. Although this initiative is commendable, the need for country-level monitoring of the genome of parasite populations for possible evolution and selection due to drug pressure is also crucial for the early detection of emerging drug resistance.

For over a decade, molecular markers of antimalarial drug resistance have been used to monitor the emergence and spread of drug resistance in malaria endemic areas. These genetic markers are mainly single nucleotide polymorphisms in genes encoding drug target proteins in essential biochemical pathways of the parasite. The levels of drug susceptibility in the parasites have been linked to SNPs or haplotypes of the genes, and these markers are relevant in antimalarial drug efficacy studies. The recent observation of parasite resistance to ART in SEA set into motion the need to discover a molecular marker for surveillance of drug susceptibility. Ariey et al. [3] discovered the SNPs in the kelch propeller domain on chromosome 13 of the *P. falciparum* genome known as k13 in drug resistance isolates *in vitro*. Three of the k13 polymorphisms, C580Y, R538T and Y493H were also present in slow clearing clinical isolates with ART use. The presence of the SNPs showed varying parasite clearance half-life in patients; however, the C580Y mutation was linked to longest parasite clearance half-life compared to the other SNPs. So far these SNPs have not been detected in African isolates [4–6] and the quest for novel markers for ART resistance is ongoing. Henriques et al. [7] linked SNPs in two genes, the *P*. *falciparum* adaptor protein complex 2 mu subunit (*pfap2mu*) and ubiquitin-specific protease 1 (*pfubp1*) to delayed clearance of parasites. The *pfap2mu* gene mutation was at codon 160 resulting in the amino acid change from serine to either asparagine or threonine (S160N/T). The *pfubp1* gene mutations were at codon 1525, a change from aspartic acid to glutamic acid (D1525E) and codon 1528 from glutamic acid to aspartic acid (E1528D) in the Kenyan isolates. In addition, Sutherland et al. [8] reported four UK residents with imported malaria who showed recurrent malaria after AL treatment [8]. These mutations, *pfap2mu* S160N and *pfubp1* E1525D/Q were observed in the recurrent parasites but none of the known K-13 gene mutations were observed [8]. Although the role played by both genes in artemisinin action is not clearly understood, the *ap2mu* gene is known to encode the μ- subunit of the adaptor protein 2 complex (AP2) involved in clathrin-mediated endocytosis into the parasite vacuole [9]; *ubp1* encodes a deubiquitinating (DUB) enzyme that functions by cleaving ubiquitin from any protein or peptide to which it is joined [10]. The polymorphic homologues of these two *P. falciparum* genes were first identified in the rodent malaria parasite, *P*. *chabaudi* (*pcubp1* encodes ubiquitin carboxy-terminal hydrolase 1) and *pcap2mu* encodes clathrin vesicle-associated adaptor 2 mu subunit), as being associated with ART resistance [11]. More studies are therefore needed to validate these polymorphisms and their role in antimalarial drug resistance.

The use of ACTs in Ghana began in 2005 and since then the cure rate of the drugs in use, artesunate-amodiaquine (AS-AQ) and artemether-lumifantrine (AL), has been 100 and 97.6%, respectively, as of 2014 [12]. Surveillance studies using the *P. falciparum* multidrug resistance gene (*pfmdr1*) SNPs (haplotype N86-F184-D1246) linked to reduced parasite susceptibility to AL showed an increasing trend over the years in Ghana from 2005 to 2010 [13]. In addition, increased *pfmdr1* gene copy number linked to parasite reduced susceptibility to artesunate (AS), mefloquine (MQ), halofantrine and AL [14–16] were also detected in Ghanaian isolates [13]. The findings from the reported studies above are indicative of a subtle emergence of parasite resistance to ART and to ACTs especially AL in Ghana. Therefore, the monitoring of newly discovered molecular markers is essential as an early warning signal to the emergence of resistance in Ghana. This study determined the prevalence of known and novel SNPs in the *pfubp1* and *pfap2mu* in Ghanaian isolates collected from three ecologically distinct areas for monitoring antimalarial drug efficacy in Ghana to serve as baseline data for antimalarial drug resistance surveillance in Ghana.

## Methods

### Study sites

**Fig. 1:**
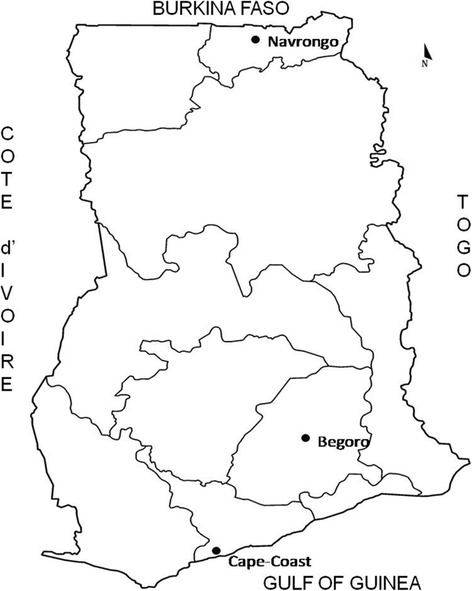
The map of Ghana showing the three study sites, Navrongo, Begoro and Cape Coast in three different ecological areas, guinea savannah, forest and coastal savanna

The Noguchi Memorial Institute for Medical Research (NMIMR) in collaboration with the National Malaria Control Programme (NMCP) have set up ten sentinel sites in the ten regions of Ghana for monitoring antimalarial drug efficacy. These sites lie in the three distinct ecological zones in the country. Of the ten sites, samples from three sites were used for this study. The sites include Navrongo (10°53'44.05"N, 1°05'31.56"W) located in the Kassena Nankana District in the Upper East Region and lies in the guinea savannah zone; Begoro (6°23'29.76"N, 0°22' 46.20"W) located in the Fanteakwa District of the Eastern Region lies in the forest zone; Cape Coast (5°06'00”N, 1°15'00"W), the capital town of Central Region lies in the coastal savannah zone (Fig. 1). The forest and coastal savannah zones experience perennial malarial transmission whilst the guinea savannah zone experiences a seasonal malaria transmission pattern with almost all cases occurring during rainy months between May-June and October-November of each year.

### Study samples

Filter paper blood blots collected in 2015–2016 from children aged below 9 years with uncomplicated malaria reporting at designated health care facilities in Navrongo, Begoro and Cape Coast were used for the study. In total 120 samples (40 from each site) were used for this investigation.

### Detection of *pfubp1* and *pfap2mu* gene polymorphisms

Parasite DNA was extracted from 120 pre-treatment blood blot samples on filter paper (Whatman^TM^ 3 Little Chalfont, United Kingdom) using the QIAmp DNA mini kit (Qiagen GmBH, Hilden, Germany) as per the manufacturer’s protocol. This was followed by amplification of the *pfap2mu* and *pfubp1* genes using nested polymerase chain reaction (nPCR) following a previously published protocol [7] with minor modifications. The PCR was performed in a total volume of 25 μl with the following reaction mixture: 0.2 μM of each primer (Table 1), 4.0 mM MgCl_2_, 0.4 μM deoxynucleotides triphosphate (dNTPs), 1 U One-Taq polymerase (New England Biolabs, Massachusetts, USA), 1× PCR buffer, nuclease free water and 2 μl of the extracted parasite DNA. One microlitre of the first round product was used as a template in a 50 μl inner PCR reaction. A DNA sample extracted from the 3D7 parasite strain was used as a positive control. The PCR thermal conditions were the same for both genes but different annealing temperatures as shown in Table 1. The thermal cycle programme for each 1st amplification was 94 °C for 3 min, and 30 cycles of 94 °C for 30 s, annealing temperature for 30 s and 68 °C for 1 min with a final extension of 68 °C for 15 min. The second round of PCR consisted of initial denaturation at 94 °C for 3 min, followed by 40 cycles of 94 °C for 30 s, annealing temperature for 30 s and 68 °C for 45 s with a final extension of 68 °C for 10 min. The PCR amplicons for the fragments of the two genes were sequenced using Sanger sequencing.

**Table 1:** Primer sequences, sizes of PCR amplicons and annealing temperature of the amplification of *pfap2mu* and *pfubp1* genes

Gene	Primers (5'–3')	Size of PCR amplicon (bp)	Annealing temperature (°C)
*pfap2mu*	Primary amplification	2247	50
	Forward: AAGACTGTCAAATGTAAAAGACCC		
	Reverse: CTCATGTAAAACAAAAAGTGAGG		
	Secondary amplification	841	52
	Forward: GATATCCACAAACATTAGAAGTG		
	Reverse: CCATCTGGTGGTGTGAAGG		
*pfubp1*	Primary amplification	484	52
	Forward: CGCCCGTACTATGAAGAAGATC		
	Reverse: GGCTTTTACCTGAACTGTTCAGG		
	Secondary amplification	304	57
	Forward: CGTAAACAGAATATTCAGGATTGC		
	Reverse: CTAGCCCTTTATTATCATTATCG		

### Data analysis

The sequence data of the isolates were analysed using the CLC Genomics Workbench 10.01 software (Qiagen, Aarhus, Denmark) and Benchling.com (California, CA, USA). PF3D7_1218300 and PF3D7_0104300 (PlasmoDB) were used as reference sequences to detect SNPs in the *pfap2mu* and *pfubp1* respectively. Poor quality sequences of isolates after three sequencing trials were not analysed. The prevalence of individual SNPs was determined for each site. Chi-square tests were used to compare the proportions of mutations occurring in the three sites and to determine any significant differences in the prevalence of the mutations among the three sites using the GraphPad Prism 5 (GraphPad Software Inc, La Jolla, CA, USA). Statistical significance was defined as a *P-*value ≤ 0.05.

## Results

### Polymorphisms in *pfap2mu* and *pfubp1*

**Fig. 2:**
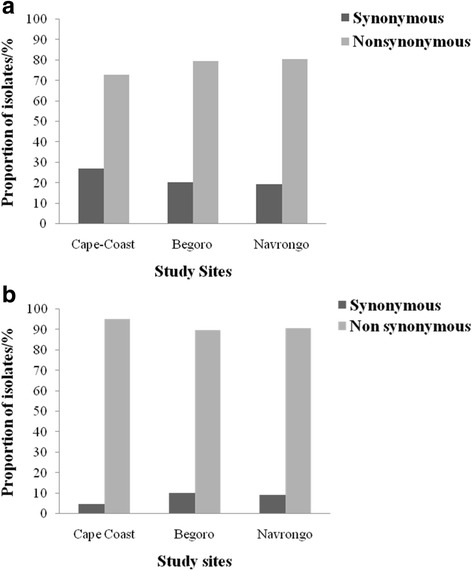
Distribution of *pfap2mu* and *pfubp1* NS and SYN mutations in isolates from the three sites. **a** *pfap2mu.* **b** *pfubp1*

**Fig. 3:**
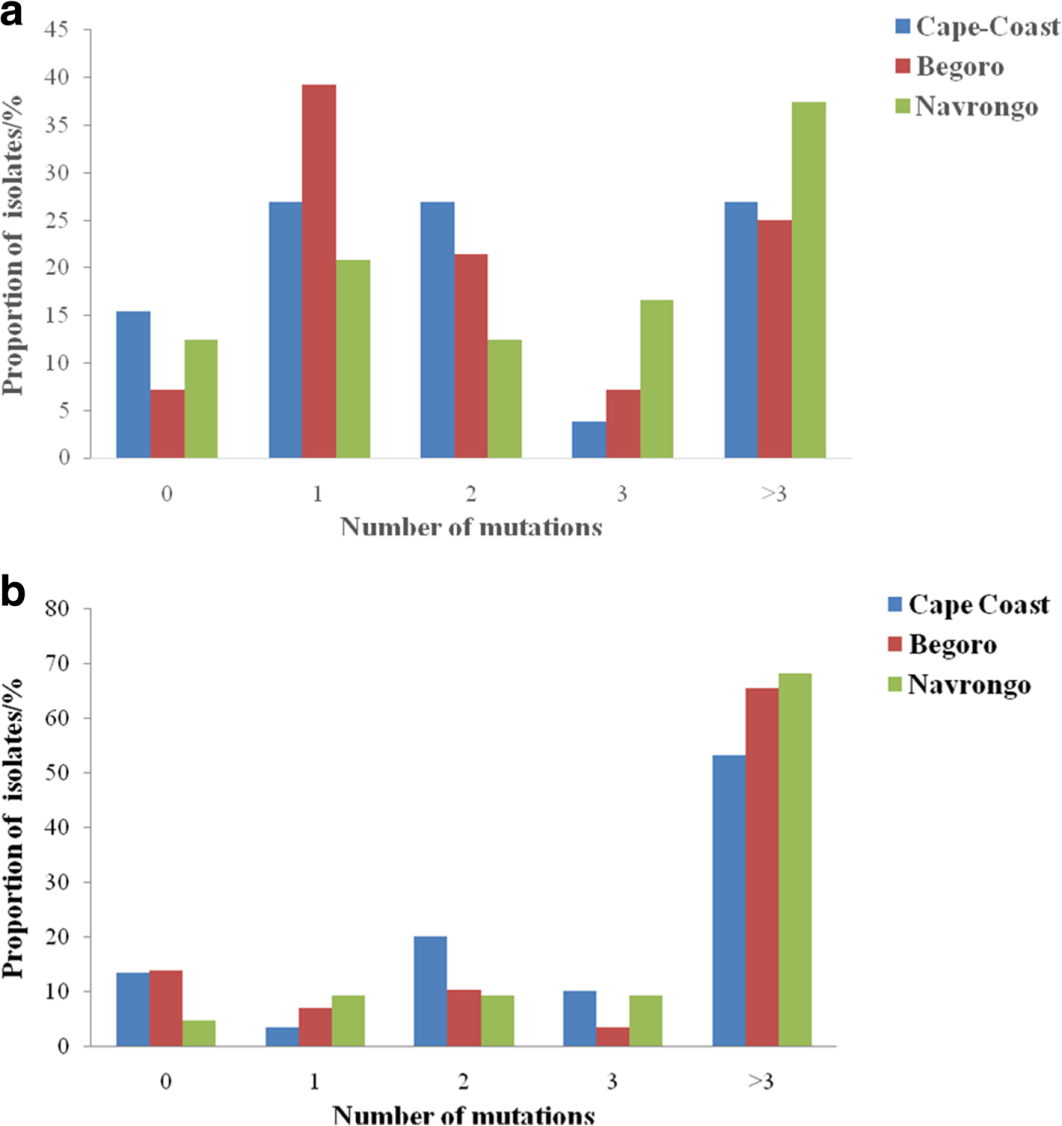
Proportion of isolates from the three sites with varying number of *pfap2mu* and *pfubp1* mutations. **a** *pfap2mu.* **b** *pfubp1*

**Fig. 4:**
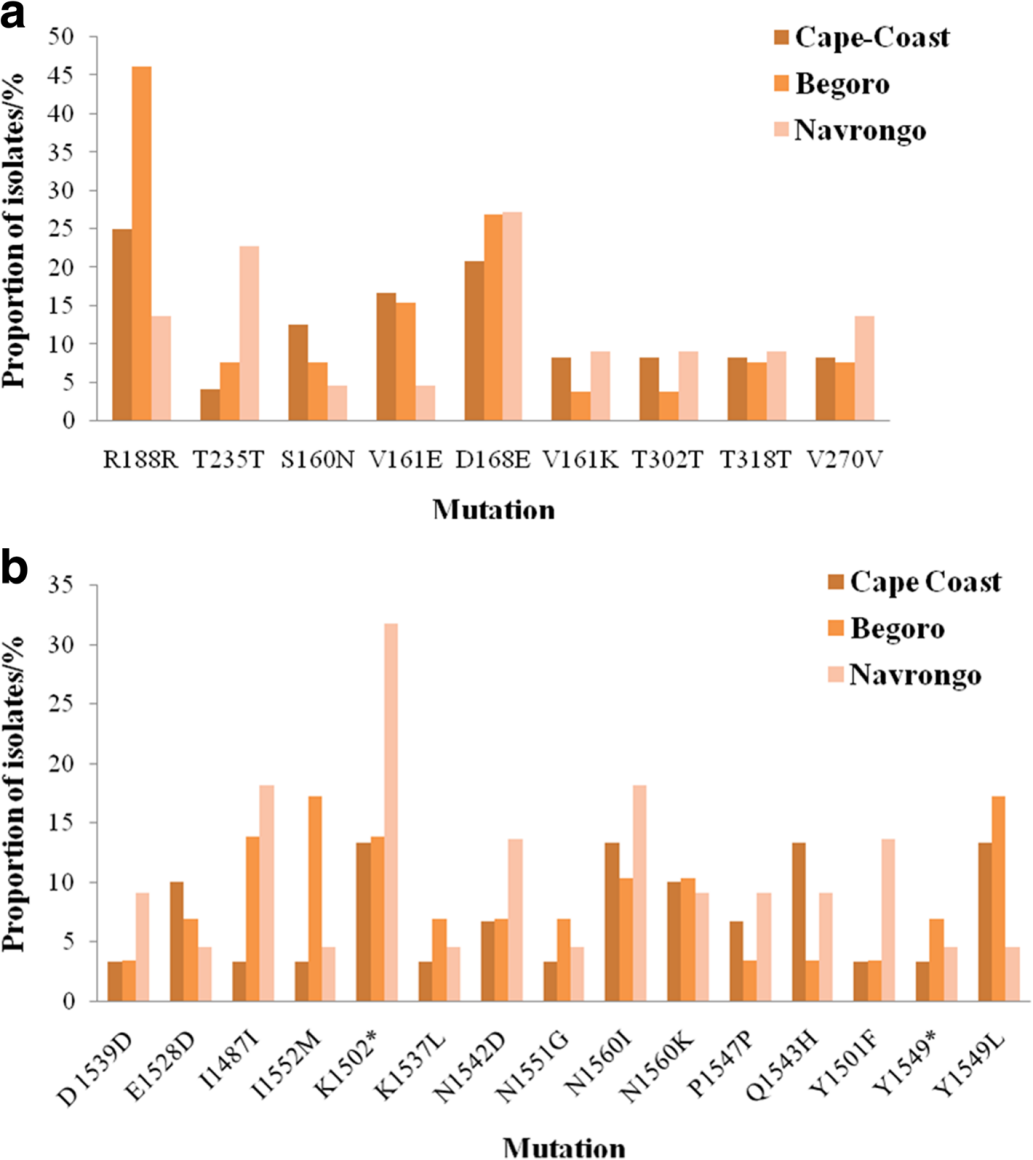
Proportion of isolates from the three sites with shared *pfap2mu* and *pfubp1* mutations. **a** *pfap2mu.* **b** *pfubp1*

**Fig. 5:**
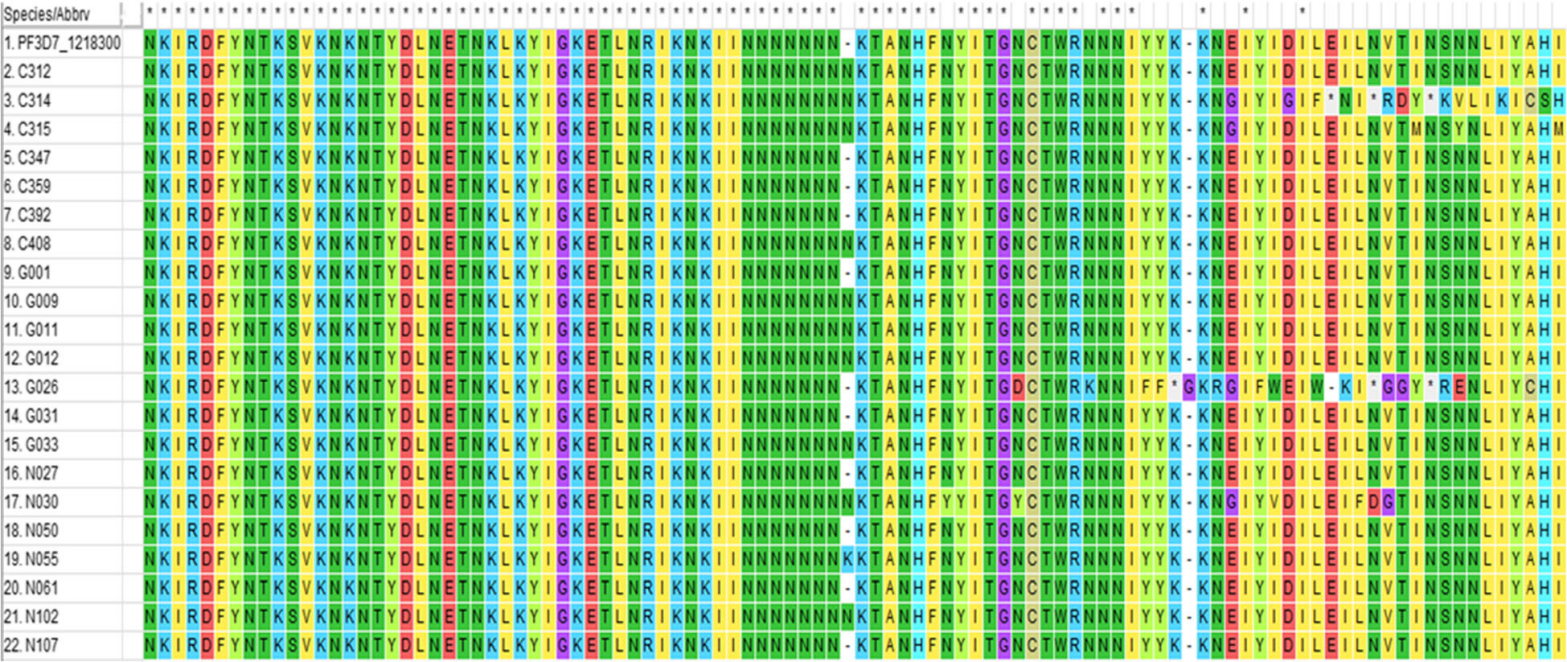
A sequence alignment of *pfap2mu* gene showing amino acid changes due to single nucleotide polymorphisms. The alignment was done using *pfap2mu* reference sequence of the 3D7 strain (PF3D7_1218300). Mutations present at codons 185–282, nucleotide positions 553–849 of *pfap2mu for* 21 samples. Samples C314 and G026 had a frameshift, samples C312. C314, C315, C408, G009, G011, G012, G033, N030 and N102 had an asparagine (N) insertion at codon 233, as well as a lysine (K) insertion at the same position for sample N055

**Fig. 6:**
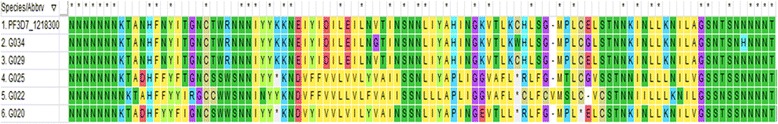
A sequence alignment for 5 samples with multiple mutations. The alignment was done using *pfap2mu* reference sequence of the 3D7 strain (PF3D7_1218300). About 215 SNPs were observed in these isolates ranging from 15 to 63 SNPs per isolate. These mutations were found from codons 227–324, nucleotide positions 679–970 and are likely due to multiplicity of infection

For the *pfap2mu* gene, 96 samples were sequenced and 15 were of low quality as determined by quality assurance analysis. Of the 81 good sequences, 35% (28/81), 35% (28/81) and 31% (25/81) were from Cape-Coast, Begoro and Navrongo, respectively. The proportion of isolates with no mutations in the *pfap2mu* gene, that is wildtype sequence as the 3D7 strain, was 11.1% (9/81). The sequence analysis revealed several SNPs and the total number observed in 67 samples (of the 72 with mutations) was 164 SNPs with ≤ 5 mutations per sample. Of the 164 SNPs, 78.0% (128/164) were non-synonymous (NS) and 22.0% (36/164) were synonymous (SYN) mutations. The distribution of *pfap2mu* NS and SYN mutations in isolates from the three sites is shown in Fig. 2a. There was no significant difference in the type of mutation (SYN or NS) present between the three sites (*χ*^2^ = 1.960, *df* = 2, *P* = 0.360). The distribution of the single or multiple mutations for each site is shown in Fig. 3a. About 68.6% (46/67) of the isolates with mutations had more than one mutation and 36% (9/25) of the isolates from Navrongo had more than three mutations per isolate. In addition, there were insertions and deletions in some of the isolates from the three sites. In all 22 common SNPs were detected in either two of the three sites or all three sites. These include Q149R, S160N, V161K, V161E, D168E, R188R, D203F, D203Y, E206*, T235T, N240Y, N240F, K256*, D263V, V270V, I272I, G284G, K285E, T302T, N317S, T318T and T325T. Nine of these SNPs were shared in all three sites and the proportion of isolates from the study sites is shown in Fig. 4a. Of these SNPs, three NS mutations were present in isolates from the three sites: S160N, D168E and V161K. The nucleotide changes for the SNPs are shown in Table 2. The amino acid sequences alignment for 21 isolates are shown in Fig. 5. There were 5 other isolates (of the 72 with mutations) with a total of 215 *pfap2mu* SNPs (G020, 63 SNPs; G022, 60 SNPs; G025, 47 SNPs; G029, 30 SNPs; G034, 15 SNPs) and were all from Begoro. The amino acid sequences for these 5 isolates are shown in Fig. 6.

**Table 2:** Shared *pfap2mu* mutations observed in the isolates from the three sites. Mutations indicated in bold are known delayed clearance genotypes, underlined nucleotides are the changed bases

Nucleotide position	Nucleotide change	Amino acid position and change
446	CAG to CGG	Q149R
479	AGT to AAT	**S160N**
481	GTG to AAG	V161K
482	GTG to GAG	V161E
504	GAT to GAA	D168E
564	AGA to AGG	R188R
607	GAT to TAT	D203Y
607, 608	GAT to TTT	D203F
616	GAA to TAA	E206^a^
705	ACA to ACG	T235T
718	AAT to TAT	N240Y
767	AAG to TAG	K256^a^
789	GAT to GTT	D263V
810	GTA to GTT	V270V
852	GGA to GGG	G284G
855	AAG to GAG	K285E
951	AAC to AGC	N317S
954	ACA to ACC	T318T

^a^Stop codon

**Fig. 7:**
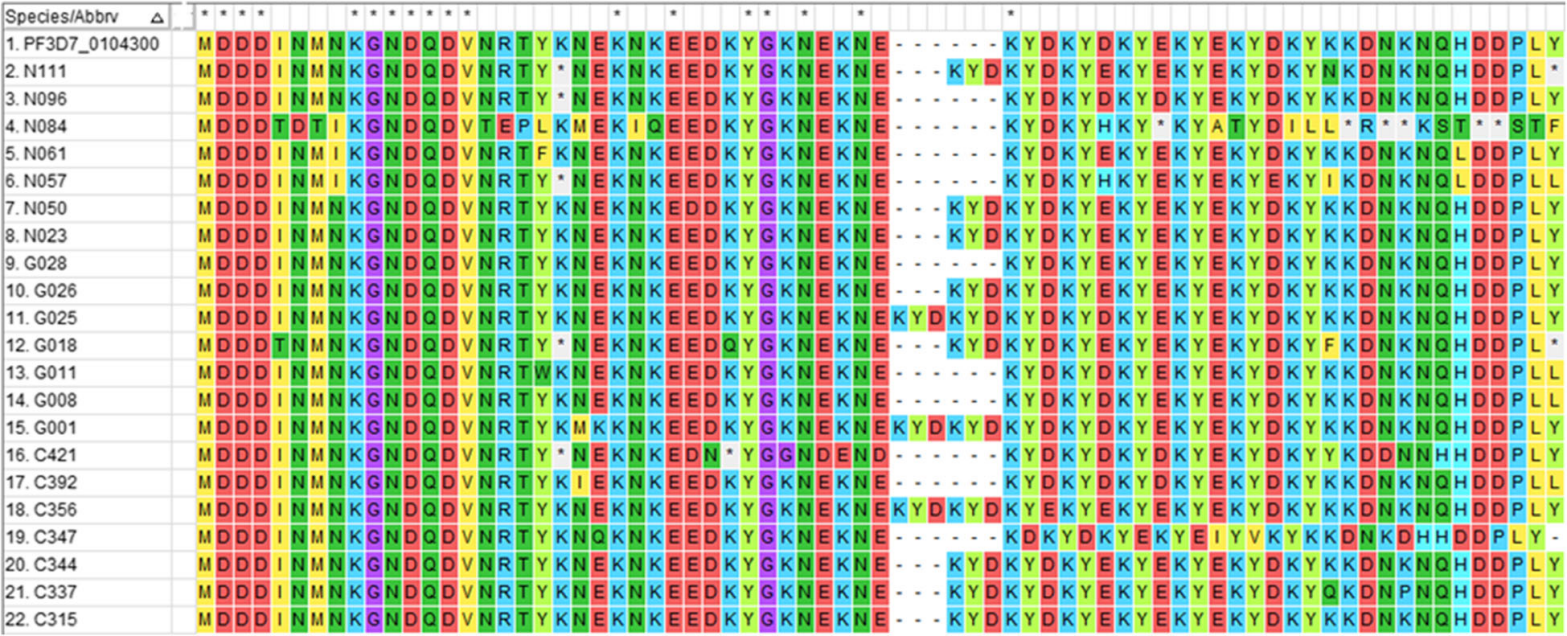
A sequence alignment of *pfubp1* gene showing amino acid changes due to single nucleotide polymorphisms. The alignment was done using *pfubp1* reference sequence of the 3D7 strain (PF3D7_0104300). Mutations from codons 1483–1549 at nucleotide positions 4449–4647 is shown. The gaps are a result of the insertions of amino acids which resulted in a frameshift. The gap between 1519E and 1520K are therefore a result of a 6 amino acid insertion in C356, G001 and G025. The known mutations D1525E and E1528D are shown in the isolates N061 and N096 respectively. A frameshift mutation is observed in N084, after codon 1536 as a result of a deletion

For the *pfubp1* gene, 81 quality sequence data were analysed comprising 37% (30/81), 35.8% (29/81) and 27.2% (22/81) from Cape Coast, Begoro and Navrongo, respectively. About 11.1% (9/81) of the isolates had no mutations in their *pfubp1* gene. A total of 271 SNPs were observed in the 72 sequences of which 92.3% (250/271) were NS and 7.7% (21/271) were SYN mutations. The proportion of isolates with either *pfubp1* NS or SYN mutations for each site is shown in Fig. 2b. The proportion of isolates with varying number of mutations is also shown in Fig. 3b for the three study sites. Overall, 93.1% (67/72) of the isolates with mutations had more than one *pfubp1* SNP. The majority of isolates from Begoro (65.5%, 19/29) had more than 3 mutations per isolate for the *pfubp1* gene. There were 15 common SNPs detected in isolates from all three sites. These include D1539D, E1528D, I1487I, I1552M, K1502*, K1537L, N1542D, N1551G, N1560I, N1560K, P1547P, Q1543H, Y1501F, Y1549* and Y1549L. The proportion of isolates with these mutations from each of the study sites is shown in Fig. 4b. There were 36 other shared SNPs detected in isolates from two out of the three sites. The nucleotide changes for the commonly shared mutations are also shown in Table 3. The amino acid sequence alignment is shown in Fig. 7.

**Table 3:** Shared *pfubp1* mutations observed in the isolates from the three sites. Mutations indicated in bold are known delayed clearance genotypes

Nucleotide position	Nucleotide change	Amino acid position and change
4383	CCT to ACC	P1461T
4386	TAT to TTA	Y1462L
4389	CGT to TCG	R1463S
4392	AAA to TAA	K1464^a^
4461	ATA to ATC	I1487I
4461	ATA to ACC	I1487T
4466	ATG to ACG	M1489T
4502	TAT to TTT	Y1501F
4504	AAA to TAA	K1502^a^
4509	AAT to ATT	N1503I
4509	AAT to ATG	N1503M
4527	GAA to GAC	E1509D
4554	AAC to TAT	N1518Y
4557	GAA to GAC	E1519D
4561	TAT to AAT	Y1521N
4575	GAC to GAA	**D1525E**
4581	TAT to TTT	Y1527F
4581	TAT to AAT	Y1527N
4584	GAA to GAC	**E1528D**
4599	TAT to TAC	Y1533Y
4602	GAT to GAA	D1534E
4608	TAC to TCC	Y1536S
4611	AAA to TTT	K1537F
4609	AAA to TTA	K1537L
4611	AAA to TAC	K1537Y
4617	GAT to GAC	D1539D
4623	AAA to CTT	K1541L
4623	AAA to AAT	K1541N
4626	AAT to GAT	N1542D
4629	CAA to CAT	Q1543H
4632	CAT to CTT	H1544L
4632	CAT to CCT	H1544P
4632	AAA to AAT	K1544N
4641	CCA to CCT	P1547P
4647	TAT to TAG	Y1549^a^
4647	TAT to TTT	Y1549F
4646	TAT to TTG	Y1549L
4650	GAT to ATT	D1550I
4650	GAT to AAT	D1550N
4651	AAT to GGT	N1551G
4653	AAT to ATT	N1551I
4653	AAT to CTT	N1551L
4656	ATT to ATG	I1552M
4659	AAT to AAC	N1553N
4667	TAC to TGC	Y1556C
4674	AAT to GAT	N1558D
4679	AAT to ATA	N1560I
4680	AAT to AAA	N1560K
4683	AAA to AAG	K1561K
4692	GAG to GAC	E1564D
4695	TTC to GGC	F1565G
4694	TTC to CAA	F1565Q
4765	AAA to TAA	K1589^a^

^a^Stop codon

Two samples, G005 and C329, had wildtype sequences for both *pfap2mu* and *pfubp1*. Of the 5 isolates with many *pfap2mu* SNPs, 2 were wildtype for *pfubp1* (G022 and G034). For the other three, G020 had 4 SNPs, G025 had 2 SNPs and G029 had 11 SNPs for the *pfubp1*.

### Prevalence of *pfap2mu* and *pfubp1* SNPs from the three sentinel sites

The *pfap2mu* SNPs were detected in 92.9% (26/28), 85.7% (24/28) and 88% (22/25) from Begoro, Cape Coast and Navrongo respectively. There was no significant difference in the prevalence of *pfap2mu* SNPs in the isolates from the three sites (*χ*^2^ = 6.905, *df* = 2, *P* = 0.546). The most prevalent mutation was R188R and was observed in 25.9% (21/81) of the isolates. The D168E was also observed in 22.2% (18/81) of the isolates. S160N which have been reported to be associated with ACT delayed clearance was prevalent in 7.4% (6/81) of the isolates whilst the V161K was seen in 6.2% (5/81). The S160N genotypes occurred mostly in isolates from Cape Coast (24.7%) as compared to the other two sites. In all, 35 indels were identified with 17.1% (6/35) causing a frame shift in the sequence reading frame (Fig. 5).

For *pfubp1*, mutations were detected in 86.7% (26/30) of the Cape Coast isolates, 86.2% (25/29) for Begoro and 95.5% (21/22) for Navrongo. There was no significant difference in the prevalence of SNPs in the isolates from the three sites (*χ*^2^ = 4.883, *df* = 2, *P* = 0.769). The most prevalent NS mutation was K1502* which was observed in 18.5% (15/81) of the isolates. The reported SNPs linked to delayed clearance of parasite with ACT use, E1528D and D1525E, were observed in 7.4% (6/81) and 4.9% (4/81) of the isolates, respectively. The predominant SYN mutation I1487I was observed in 11.1% (9/81) of the isolates. In all, 20 indels were identified with 25% (5/20) causing a frame shift in the sequence reading frame (Table 4).

**Table 4:** Genetic insertions in the *pfubp1* gene of isolates from the study sites

Sample ID	Amino acids	Insertion	Nucleotide position
C315	KYE	AAA TAT GAA	4576 to 4584
C337	KYE	AAA TAT GAA	4583 to 4591
C344	KYE	AAA TAT GAA	4548 to 4556
C356	KYEKYE	AAA TAT GAA AAA TAT GAA	4588 to 4605
C360	KYE	AAA TAT GAA	4583 to 4591
C321	EKY	GAA AAA TAT	4590 to 4598
G001	DKYDKY	GAC AAA TAT GAC AAA TAT	4563 to 4580
G018	EKY	GAA AAA TAT	4598 to 4606
G025	KYDKYE	AAA TAT GAC AAA TAT GAA	4585 to 4602
G026	EKY	GAA AAA TAT	4580 to 4588
G034	YDKYDK	TAT GAC AAA TAT GAC AAA	4579 to 4596
N023	EKY	GAA AAA TAT	4582 to 4590
N031	EKY	GAA AAA TAT	4582 to 4590
N050	KNE	AAA AAC GAA	4549 to 4557
N111	YEK	TAT GAA AAA	4597 to 4605

The results showed a number of SNPs that were being inherited together on the gene as haplotypes in some of the isolates. However, the prevalence of the haplotypes was low (Table 5)**.** Twenty-two different haplotypes were observed: 3 for *pfap2mu* and 19 for *pfubp1*. The most prevalent haplotype for the *pfap2mu* gene was V161K-D168E, which was observed in 8.6% (7/81) of the isolates with mutations. For the *pfubp1* gene, the haplotypes Y1548L-N1560I, Y1549L-I1552M and N1560I-L1563 were each observed in 3.7% (3/81) of the isolates. Most of the haplotypes for both genes were observed in isolates from Begoro.

**Table 5:** Haplotypes of the *pfap2mu* and *pfubp1* mutations in Ghanaian isolates

Gene	Haplotype	No. of isolates
*pfap2mu*	V161K-D168E	7
	V161E-R188R	6
	D168E-R188R	6
*pfubp1*	I1487I-N1488D	2
	I1487I-N1490I	2
	Y1549L-I1552M	3
	N1540H-K1541N	2
	N1540H-H1544P	3
	N1540H-N1542D	2
	N1488D-D1525H	2
	I1487I-Y1501F	2
	N1490I-Y1501F	2
	N1560I-L1563A	3
	E1528D-N1560K	2
	Y1549L-N1560I	3
	Y1549L-I1552M	2
	Y1501W-Y1549L	2
	N1542D-Q1543H	2
	N1488D-G1492G	2
	Q1543H-P1547P	2
	I1487I-N1490I-H1544L	2
	N1490I-Y1501F-Y1533Y-P1547P-K1554N-N1555I-D1557N	2

## Discussion

The search for a molecular marker for the early detection of ART resistance by the malaria parasite is ongoing. The use of molecular markers to track and identify early development of parasite resistance to drugs is a powerful tool that should be available in all malarious regions of the world. With the implementation of ACTs in Africa, studies to identify possible markers of resistance have not been conducted extensively in the continent. The key *k13* molecular marker, which has been linked to drug resistance in SEA has not been observed in African isolates, partly because there is no ‘true resistance’ to ARTs except delayed clearance of parasites. New markers that have recently been discovered, such as the *pfap2mu* and *pfubp1* gene mutations [7], need further validation for their role in delayed parasite clearance. This study detected ART delayed clearance associated polymorphisms of the *pfap2mu* and *pfubp1* genes in Ghanaian isolates from three sites located in three distinct ecological zones. Majority of the isolates had mutations of both genes and were mostly NS mutations. The known delayed clearance genotypes, *pfap2mu* N160 and *pfubp1* D1528 and E1525, were observed in 7.4, 7.4 and 4.9% of the isolates, respectively. It is interesting to note that although these were not the predominant mutations, it is an indication of the presence of these drug resistance genotypes in Ghanaian parasite populations and in the long term their selection with drug use will enhance the emergence of ART resistance in Ghana.

The observation that minority of the isolates were wildtype with no mutations like the reference 3D7 strain (11.1% for both *pfap2mu* and *pfubp1*) is indicative of the high rate of spontaneous mutations in the two genes. For *pfap2mu*, a study by Henriques et al. [7] reported that 41.5% of Kenyan isolates had wildtype gene sequence whilst another study reported 92.8% and 30.9% for Ethiopian and Tanzanian isolates, respectively [17]. Comparatively, the Ghanaian isolates had low levels of wildtype strains portraying a rapid genetic recombination of different parasite clones (multiclonal infections observed in Ghana) during the sexual stage in the vector resulting in gene shuffling [18]. About 64% and 93% of the isolates, respectively, had more than one mutation for the *pfap2mu* and *pfubp1* genes.

The results revealed a high proportion of NS mutations, 78% and 92% for *pfap2mu* and *pfubp1*, respectively, in the Ghanaian isolates. The NS mutations are of much importance because each amino acid substitution may affect protein conformation and function [19]. Most of the NS mutations were single base or double base substitutions. For SYN mutations, it was initially assumed that since the resultant change of the nucleotide does not affect the amino acid, the change may go undetected as the gene function may not necessarily be affected [20]. However, this perception has since changed due to the evidence that SYN mutations in the *pfmdr1* gene resulted in alterations in the functions of the P-glycoprotein (P-gp), a product which affects drug interactions [21]. A high prevalence of novel *ap2mu* mutation, D168E (25%), was observed in the Ghanaian isolates from all three sites followed by V161K (7%). The codons for the common mutations found among the isolates from all the three sites were between codons 160 and 170. The known SNP, *pfap2mu* S160N, found in Kenyan isolates with delayed clearance [7] was also found in all three sites (7.2%). In addition, isolates from a UK patient who arrived from Angola and failed AL treatment had the S160N thereby strengthening the role of that mutation in recurrent parasitemia with AL use [8]. The observed *pfubp1* E1528D in Ghanaian samples was also seen in Kenyan and Tanzanian isolates [7, 17]. Borrmann et al. [22] first identified this mutation in Kenyan isolates. The prevalence of E1528D in Ghanaian isolates was 7.4%, which is lower than that of Kenya’s 17.1% but higher than that of Tanzania (4.8%) [7, 17]. However, with the continuing use of ACTs, the mutation may be selected and the prevalence may increase with time as observed in Kenya. The SYN mutation N1518 found in the Kenyan and Burkinabe isolates was also seen in one of the Ghanaian isolates from Begoro. Pre- and post-treatment samples from Burkina Faso, had the D1525E mutation which was seen in four Ghanaian isolates. Henriques et al. [7] compared South East Asian and African phenotypes of the *pfubp1* gene and detected significant differences in the genetic signatures. The recent implication of SNPs in these genes, especially *pfap2mu* S160N and *pfubp1* E1528D and D1525E in ART resistance, raises a genuine concern due to their presence in Ghanaian isolates.

Of the three sites, Begoro (forest) had the most *pfap2mu* mutations (93%) followed by Cape-Coast (coastal savannah) (86%) and Navrongo (guinea savannah) (88%). Despite this observation, there is comparatively higher diversity in the mutations in Navrongo isolates with 72 different SNPs. Most of the isolates (63%) had more than one mutation. The high diversity of SNPs in Navrongo, where there is intense seasonal transmission of malaria, is expected. For *pfubp1*, Navrongo had the most mutations (96%), followed by Cape Coast (87%) and Begoro (86%). It is quite interesting to observe diversity of mutations from both genes from the guinea savannah zone. However, it must be emphasised that transmission intensity does not affect the evolution of resistance but plays a major role in the spread of resistance genotypes [18]. Therefore, discussing our observations along the line of different transmission intensities from the three distinct ecological zones will be premature.

Insertions/deletions (indels) identified in the Ghanaian isolates were similar to those observed in Burkina Faso, Kenya and the UK patients from Liberia and Uganda [7, 8]. The most common indels, observed in the isolates from the Ghanaian and the other African countries resulted from an insertion of AAT which resulted in an asparagine and lysine residues at codons 226 and 233, respectively [7, 8]. Indels of one to six amino acids were observed for the *pfubp1* gene in the Ghanaian isolates and these were similar to those seen in Kenya and Burkina Faso [7, 8]. These resulted from an insertion of a KYD, KYE or KNE amino acids at codons ranging from 1516 to 1535. Most of the indels that resulted in a frameshift were caused by a deletion or insertion of either a guanosine or thymidine nucleotide.

The presence of the molecular markers implicated in drug resistance is of great importance in their role in modulating drug susceptibility and subsequently the prediction of the dynamics of resistance [23]. The spread of *P*. *falciparum* resistance to ARTs is a real global challenge and therefore insights into the mechanisms of drug action and resistance are critical for early detection of resistance. The detection and characterisation of these mutations in post-treatment Ghanaian isolates is the way forward for further validation of their roles in conferring resistance. This study has, therefore, highlighted the existence of delayed clearance markers of the *pfap2mu* and *pfubp1* genes in circulating parasites in Ghana and will serve as baseline data for future surveillance studies.

## Conclusions

The identification of molecular markers of ART resistance in Ghanaian isolates has implications for the development of ACT resistance especially for AL use. The findings from this study give first-hand information on potential molecular markers of ART resistance in Ghana and as such highlight the possibility of circulating parasites with reduced susceptibility to ACTs in use. Further investigations are underway to ascertain their contribution to the less than 100% cure rates observed, particularly for AL.

## Additional file

Additional file 1: **Table S1.** The list of all observed *pfap2mu* SNPs in Ghanaian isolates from the three study sites. **Table S2.** The list of *pfap2mu* SNPs from five isolates with at least 20 SNPs per sample. **Table S3.** The list of all observed *pfubp1* SNPs in Ghanaian isolates from the three study sites. (XLSX 41 kb)

## Abbreviations

ACT: artemisinin-based combination therapy
AL: artemether-lumifantrine
ART: artemisinin
AS-AQ: artesunate-amodiaquine
DUB: deubiquitinating enzyme
IRB: Institutional Review Board
IRS: indoor residual spraying
ITNS: insecticide-treated mosquito nets
NMCP: National Malaria Control Programme
nPCR: nested polymerase chain reaction
NS: non-synonymous mutations
*pfap2mu*: *P. falciparum* adaptor protein complex 2 mu subunit gene
*pfk13*: *P. falciparum* kelch propeller domain on chromosome 13
*pfubp1*: *P. falciparum* ubiquitin specific protease 1 gene
SEA: Southeast Asia
SNPs: single nucleotide polymorphisms
SYN: synonymous mutations
WHO: World Health Organization
